# Prolactin levels and chronic kidney disease and the subsequent risk of cardiovascular events: A long term population based cohort study

**DOI:** 10.1038/s41598-025-87783-1

**Published:** 2025-02-28

**Authors:** Ehsan Rojhani, Maryam Rahmati, Faegheh Firouzi, Pardis Ziaeefar, Saber Amanollahi Soudmand, Fereidoun Azizi, Fahimeh Ramezani Tehrani, Samira Behboudi-Gandevani

**Affiliations:** 1 Reproductive Endocrinology Research Center, Research Institute for Endocrine Sciences, Shahid Beheshti University of Medical Sciences, 1985717413 Tehran, Iran; 2 Reproductive Endocrinology Research Center, Research Institute for Endocrine Sciences, Shahid Beheshti University of Medical Sciences, Tehran, Iran; 3 Urology Department, Labafinejad Hospital, School of Medicine, Shahid Beheshti University of Medical Sciences, Tehran, Iran; 4 Department of Urology, Labafi Nejad Hospital, Shahid Beheshti University of Medical Sciences, Tehran, Iran; 5 Endocrine Research Center, Research Institute for Endocrine Sciences, Shahid Beheshti University of Medical Sciences, Tehran, Iran; 6 Foundation for research & Education Excellence, Vestaria Hills, AI USA; 7 Faculty of Nursing and Health Sciences, NORD University, 8049 Bodø, Norway

**Keywords:** Cardiovascular events, Chronic kidney disease, Prolactin, Chronic kidney disease, Cardiovascular diseases

## Abstract

Both the clearance and secretion of prolactin are disrupted in chronic kidney disease (CKD). Evidence indicates that prolactin may play a role in cardiovascular (CV) disturbances. Considering the increased cardiovascular risk associated with CKD, this study investigates the relationship between prolactin levels, CKD, and the risk of CV events in both women and men, with an average follow-up period of 20 years. The study included 2,005 participants from the Tehran Lipid and Glucose Study (TLGS) who met the inclusion criteria. They were reassessed approximately every three years for a median follow-up of 19.0 years (Interquartile range (IQR):16.4–20.2), during which occurrences of CKD and CV events were recorded. A pooled logistic regression model examined the influence of Prolactin on CV events and its interaction with CKD. During follow-up, we identified 156 incident cases of CV events among men and 73 among women. Median (95%CI) PRL levels were 7.4 (5.5–10.5) ng/mL for men and 15.2 (10.3–23) ng/mL for women. The results of analyses showed that a history of CKD was associated with significantly higher odds of CV events for both men 4.2 (95% CI: 2.6–6.8) and women 5.5 (95% CI: 2.6–11.5). Results remained unchanged after adjustment for confounders including age, waist circumference, smoking, education, history of diabetes and hypertension, and family history of CV events. Interaction analyses revealed no statistically significant interaction between CKD and PRL on the odds of CV events in unadjusted and adjusted models. This consistent pattern was observed regardless of gender. Results of population-based data with over a median follow-up period of 20 years showed that CKD independently increases the risk of CV events in both men and women. However, our findings suggest that this elevated risk may not be substantially influenced by prolactin levels. Further investigation may be warranted to confirm these findings.

**Supplementary Information:**

The online version contains supplementary material available at 10.1038/s41598-025-87783-1.

## Introduction

Patients with chronic kidney disease (CKD) face an elevated risk of cardiovascular (CV) events, such as coronary artery disease and heart failure, as well as an increased risk of cardiac mortality^1,2^. CKD disrupts the hypothalamic-pituitary-gonadal axis, leading to hormonal imbalances^3^. Among these hormones, prolactin (PRL) plays a significant role. While PRL primarily regulates breast development and lactation in females^4^, its broader physiological functions, such as immune regulation, angiogenesis, and metabolic processes, are becoming clearer^5^. However, In individual with CKD, elevated serum PRL levels are well-documented, resulting from both increased production and reduced renal clearance^6^.

Emerging in vivo evidence suggests PRL contributes to CV dysfunction by affecting smooth muscle cells and endothelial cells, promoting arterial stiffness, hypertension, and end-organ damage^7–9^​. Furthermore, PRL is implicated in carotid intima-media thickness (CIMT), insulin resistance, and platelet activation, all of which can heighten the risk of CV events^10^. However, results of studies focusing on association between PRL and CV events in human, are insufficient and controversial. In a population-based study by Haring et al.^11^ involving both men and women aged 20–81 year, a positive correlation between PRL levels and increased all-cause and cardiovascular mortality has been demonstrated. In contrast, Reuwer et al.^12^in another population-based study, showed that elevated PRL levels did not predict future CV events. As such, limited research has investigated the association between PRL levels and the risk of CV events and mortality in patients with CKD. Carrero et al. (2012) suggested that PRL is associated with endothelial dysfunction, potentially increasing the risk of cardiovascular events and mortality in CKD patient^13^. Hence, in a population-based study with 20 years of follow-up, we aimed to investigate the association between PRL levels, CKD, and the risk of cardiovascular events across both genders.

## Materials and methods

### Study population

The ethics statement was issued by Shahid Beheshti University of Medical Sciences with approval number: IR.SBMU.ENDOCRINE.REC.1402.027. The informed consent was obtained both written and oral from each participant. In addition, the present study was performed in accordance with the Declaration of Helsinki.

The Tehran Lipid and Glucose Study (TLGS) is an ongoing prospective, population-based cohort study, designed to investigate the prevalence and incidence of non-communicable disease and their risk factors of among urban population of Tehran. The study population, including 15,005 men and women aged 3–69 years old, was selected through random multi-stage cluster sampling, with follow-up visits occurring approximately every three years (phase 1: 1999–2002, phase 2: 2002–2005, phase 3: 2005–2008, phase 4: 2008–2011, phase 5: 2011–2014, phase 6: 2014–2018, phase 7: 2018–2021) Further details of TLGS have been published previously^14,15^.

## Selection criteria

In the current study, 4221 subjects 20 years and older who participated in the first phase of TLGS were enrolled. After excluding those younger than 20 years old (*n* = 1,136), those with a history of CV events at baseline (*n* = 61), those with missing in PRL data (*n* = 947), those with PRL > 100 (*n* = 41), and those without any follow up visit (*n* = 31), 2005 subjects who followed until phase 7 of TLGS remained for the analyses, CKD and CV events were recorded.

## Clinical, anthropometric, and laboratory measurements

A trained physician filled out the standard questionnaire with participants’ information regarding their demographic, including age, gender, education, and physical activity, smoking habits, family history of disorders, through face-to-face interviews. The physical examination included measurements of systolic blood pressure (SBP) and diastolic blood pressure (DBP), as well as anthropometric measurements including weight (kg), height (cm), waist circumference (WC) and hip circumference (HC). Body mass index (BMI) and waist to height ratio (WHR) were calculated by dividing weight (kg) by height squared (m^2^) and WC (cm) by HC (cm), respectively. According to the standard protocol, anthropometric mismeasurements were obtained by removing shoes and wearing light clothing. A conventional mercury sphygmomanometer was used to take the average of two measurements of systolic and diastolic blood pressure in the sitting position following a five-minute rest.

Details regarding the collection of CV event data have been published in previous studies^14^. In brief, trained nurses conducted annual follow-ups with participants via phone calls to identify any CV events from the past year. If an event was reported, a trained physician collected the necessary data through home visits and/or by reviewing hospital records. The diagnosis was then confirmed by the Cohort Outcome Panel of medical specialists.

All individuals had their venous blood drawn between the hours of 7:00 and 9:00 AM, 12 to 14 h after fasting and 2 to 3 h after waking up. On the day of blood collection, all blood lipid analyses were carried out in the TLGS research facility using the Selectra 2 autoanalyzer (Vital Scientific, Spankeren, Netherlands). Serum triglycerides (TG) and total cholesterol were determined using enzymatic calorimetric techniques with glycerol phosphate oxidase, cholesterol esterase, and cholesterol oxidase, respectively. After the phosphotungistic acid-carrying lipoproteins containing apolipoprotein B precipitated, high-density lipoprotein cholesterol (HDL-C) levels were measured. Total cholesterol and HDL had intra- and inter-assay CVs of 0.5% and 2%, respectively; TG had CVs of 0.6% and 1.6%, respectively. The standard colorimetric Jaffe kinetic reaction method was used to assess the levels of serum creatinine (SCr), with sensitivity set at 0.2 mg/dl and inter- and intra-assay CVs of 2.5% and 1.9%, respectively. The assay range was 0.2–15 mg/dl, or 18–1330 mmol. The manufacturer’s recommendations for reference intervals in women and men were 53–97 mmol (0.6–1.1 mg/dl) and 80–115 mmol (0.9–1.3 mg/dl), respectively. Precinorm (cat. no. 1446070; Boehringer, Mannheim, Germany) for normal range and Precipath (cat. no. 171778; Boehringer) for pathological ranges were used as lyophilized serum controls for the assay performance evaluation after every 25 tests. Using Pars Azmoon Inc. (Tehran, Iran) kits and a Selectra 2 auto analyzer, all biochemical assays were performed. (Vital Scientific, Spankeren, The Netherlands). Prolactin (PRL) was measured by immunoradiometric assay (IRMA) (Izotop, Budapest, Hungary) using a gamma counter (Dream Gamma-10 gamma counter, Shin Jin, Korea). The intra- and inter-assay coefficients of variation for PRL were 2.3 and 3.5%, respectively. In current studies we included patients with PRL < 100 ng/ml.

## Definition

Chronic kidney disease is defined by the Kidney Disease Outcome Quality Initiative (K/DOQI) as kidney impairment or Glomerular Filtration Rate (GFR) 60 mL/min/1.73 m^2^(1.0 mL/s/1.73 m^2^) for more than 3 months. Pathologic abnormalities or damage markers, such as anomalies in blood or urine tests or imaging investigations, are considered to be indications of kidney injury^16^.

For this research, GFR was calculated using an abbreviated prediction equation derived from the Modification of Diet in Renal Disease (MDRD) study. MDRD study equation in abbreviation:

GFR = 186 × (SCr)−1.154 × (Age)−0.203 × (0.742 if female) × (1.210 if African American).

In this equation, GFR is expressed as mL/min per 1.73 m², and serum creatinine (Scr) is expressed as mg/dL.

CV events were defined as a composite measure of all occurrences of coronary heart disease (CHD), strokes, and cerebrovascular deaths. CHD was defined as heart failure, CHD death, unstable angina, angiographic proved CHD and incidences of definite and probable MI in the current investigation. A transient ischemic attack or definite or probable stroke were also included in the definition of stroke.

## Statistical method

Continuous variables were checked for normality based on the one-sample Kolmogorov–Smirnov test. They were presented as mean (standard deviation) if they had a normal distribution or median with an interquartile range (Q25-Q75) for variables with skewed distribution. Categorical variables were presented as numbers and percentages. Demographic and clinical characteristics of participants were compared according to sex status using the student t-test or chi-square test for continuous or categorical data, respectively. The Mann-Whitney test was applied to compare variables with skewed distribution.

Pooled logistic regression model was applied to assess the sex-specific association between dichotomous outcome variable and the time-dependent covariates as the data was interval censored and time to CV events was not known, and to calculate odds ratios^17^ This model treats every interval as a mini follow up study, pools the observations of all intervals together into one pooled sample and does a logistic regression on the pooled dataset. Both unadjusted and adjusted pooled logistic regression models were considered. In order to control the effect of confounding variables, variables with a P-value of less than 0.2 in the univariate analysis were added to the final multivariate models. The confounding effect of age, WC, smoking, education, history of Hypertension (HTN), Diabetes Mellitus (DM), and family history of CV events were controlled. Moreover, interaction analysis was applied to explore if the effect of PRL on the odds of the CV events is affected by CKD status. For this purpose, an interaction term of these two (PRL and CKD) was entered in the pooled logistic regression model, The odds ratios (ORs) with 95% confidence intervals (CIs) were reported in three models: Model 1: unadjusted; Model 2: adjusted for age; Model 3: Model 2 + further adjusted for WC, smoking, education, history of HTN, DM, family history of CV events. Statistical analysis was done for men and women separately. Statistical analysis was performed using software package STATA (version 13; STATA Inc., College station, TX, USA); significance level was set at *P* < 0.05, and Confidence Interval as 95%.

## Results

**Fig. 1:**
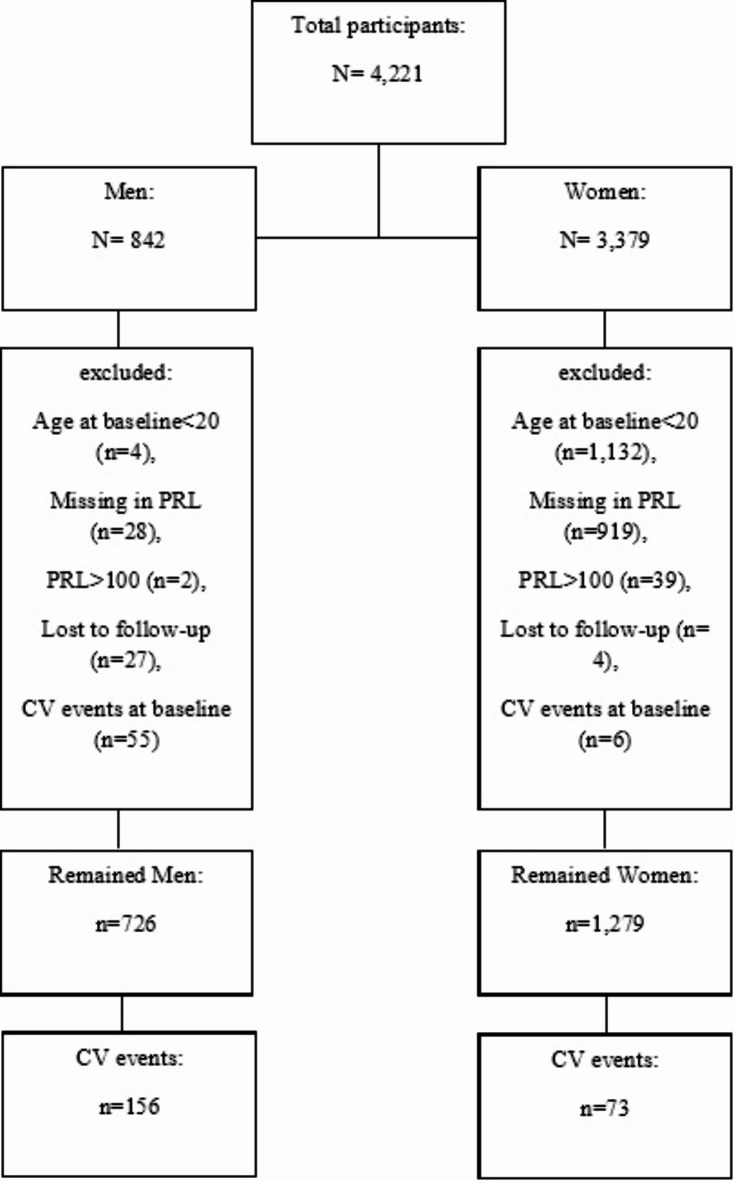
study flowchart.

Flowchart of the study is presented in Fig. 1. Among the 2,005 eligible participants, 1,279 (63.8%) were women and 726 (36.2%) were men. Baseline characteristics of the participants are presented in Table 1. The mean (SD) age of men and women were 44.5 (11.5) and 32.0 (7.4) years, respectively. The median (interquartile) for PRL was 7.4 (5.5–10.5) ng/mL for men and 15.2 (10.3–23) ng/mL for women. Mean eGFR was 75.9 (13.0) in men and 82.9 (13.4) in women. Compared to men, women were more likely to have higher levels of physical activity, less likely to be smoker, and less likely to have a history of hypertension. Compared to women, men have significantly higher history of HTN, DM, and CKD (*P* < 0.001). During a median follow-up of 19.0 years (IQR:16.4–20.2) years, we identified 156 incident cases of CV events among men and 73 among women.

**Table 1:** Baseline characteristics of participants.

variable	Total (*n* = 2005)	Men (*n* = 726)	Women (*n* = 1,279)	*p*-value
Age^a^ (year)	36.5 (10.9)	44.5 (11.5)	32 (7.4)	< 0.001
Age < 40 ^c^ (year)	1375 (68.6)	317 (43.7)	1058 (82.7)	< 0.001
Age ≥ 40^c^ (year)	630 (31.4)	409 (56.3)	221 (17.3)	< 0.001
BMI^a^ (kg/m^2^)	26.1 (4.3)	26 (4.1)	26.1 (4.5)	0.5
Waist^a^ (cm)	84.9 (11.7)	89 (11.1)	82.5 (11.4)	< 0.001
WHR^a^	0.84 (0.09)	0.92 (0.07)	0.81 (0.07)	< 0.001
WHtR^a^	0.52 (0.07)	0.52 (0.07)	0.52 (0.07)	0.5
WC^a^ (cm)	100.1 (8.6)	96.6 (7.3)	102.1 (8.6)	< 0.001
Education^c^ (upper diploma)	1130 (56.4)	393 (54.1)	737 (57.6)	0.1
Smoking^c^ (yes)	348 (17.4)	311 (42.8)	37 (2.9)	< 0.001
Physical activity^c^ (moderate to high)	591 (29.5)	150 (20.7)	441 (34.5)	< 0.001
SBP^a^ (mmHg)	113.3 (15.8)	120.1 (19.0)	109.4 (12.1)	< 0.001
DBP^a^ (mmHg)	75.3 (10.2)	78.3 (11.2)	73.5 (9.2)	< 0.001
Cr^a^	1.0 (0.18)	1.18 (0.14)	0.93 (0.12)	< 0.001
GFR^a^	79.9 (13.2)	75.8 (13.0)	82.2 (12.7)	< 0.001
EpiGFR	80.4 (13.7)	75.9 (13.0)	82.9 (13.4)	< 0.001
Prolactin^b^ (ng/mL)	11.7 (7.3–18.6)	7.4 (5.5–10.5)	15.2 (10.3–23)	< 0.001
TG^b^ (mg/dL)	122 (84–182)	155 (111–219)	106 (76–155)	< 0.001
Chol^a^ (mg/dL)	196.7 (40.5)	207.3 (39.6)	190.7 (39.7)	< 0.001
LDL^a^ (mg/dL)	124.7 (34.5)	132.2 (33.8)	120.5 (34.1)	< 0.001
HDL^a^ (mg/dL)	42.7 (10.8)	38.8 (9.5)	45 (10.8)	< 0.001
Family history of CV events ^c^	563 (27.2)	206 (26.4)	357 (27.8)	0.5
Family history of DM^c^	576 (28.7)	212 (29.2)	364 (28.5)	0.7
history of HTN^c^	109 (5.5)	65 (8.9)	44 (3.5)	< 0.001
history of DM^c^	114 (5.7)	80 (11.0)	34 (2.7)	< 0.001
CKD^c^	114 (5.7)	82 (11.3)	32 (2.5)	< 0.001

^a^ Values are presented as mean (SD), ^b^ values are expressed as median (Q_25_-Q_75_), ^c^ data shown as number (percentage).

Significant differences (P-value < 0.05), analyzed using independent samples t-test for superscripts a, Mann–Whitney U test for superscripts b and Pearson’s test for superscripts c.

BMI body mass index, WHR waist-hip ratio, WHtR waist-to-height ratio, WC waist circumference, SBP systolic blood pressure, DBP diastolic blood pressure, Cr creatinine, GFR glomerular filtration rate, TG triglyceride, Chol cholesterol, LDL low-density lipoprotein, HDL high-density lipoprotein, CV cardiovascular, CKD chronic kidney disease, HTN hypertension, DM diabetes mellitus

We first analyzed the association between PRL and CVD without adjusting for CKD. The results showed no significant relationship between PRL and CVD in both men (OR: 1.2; 95% CI: 0.9–1.0) and women (OR: 1.0; 95% CI: 0.9–1.0). Adding CKD as a covariate did not change the results (Supplementary Table 1). Table 2 presents the findings of pooled logistic regression model examining the association between PRL, chronic CKD, and CV events in both men and women. The analysis revealed the history of CKD was significantly associated with an increased risk of CV events for both men and women across all three models. In the unadjusted model, a history of CKD was associated with significantly higher odds of CV events for both men 4.2 (95% CI: 2.6–6.8) and women 5.5 (95% CI: 2.6–11.5). The significant association between CKD and CV events remained unchanged after adjusting for age. In males, the OR for CV events associated with a history of CKD was 3.4 (95% CI: 2.1–5.4), while in females, it was 4.5 (95% CI: 2.1–9.9). In the multivariable-adjusted analysis (model 3) including age, WC, smoking, education, history of HTN, DM, and family history of CV events as confounders, male individuals with a history of CKD had a 3.4 times higher risk of CV events (95% CI: 1.9–6.1; *p* < 0.001) compared to those without a history of CKD. Similarly, females with a history of CKD had a 3.0 times higher risk (95% CI: 1.4–6.4; *p* = 0.004).

**Table 2:** Pooled logistic regression model analysis for exploring the effect of CKD and PRL on CV events in men and women.

variables	Men			Women		
	Model 1	Model 2	Model 3	Model 1	Model 2	Model 3
	OR (95% CI)	OR (95% CI)	OR (95% CI)	OR (95% CI)	OR (95% CI)	OR (95% CI)
CKD	**4.2 (2.6–6.8)**	**3.4 (2.1–5.4)**	**3.4 (1.9–6.1)**	**5.5 (2.6–11.5)**	**4.5 (2.1–9.9)**	**3.0 (1.4–6.4)**
PRL (ng/mL)	1.0 (0.9–1.0)	1.0 (0.9–1.0)	1.0 (0.9–1.0)	1.0 (0.9–1.03)	1.0 (0.9–1.0)	1.0 (0.9–1.0)
CKD*PRL	0.9 (0.9–1.0)	0.9 (0.9–1.0)	0.9 (0.9–1.0)	0.9 (0.9–1.01)	0.9 (0.9–1.0)	0.9 (0.9–1.0)
Age		**1.9 (1.2–3.1)**	1.5 (0.9–2.6)		**1.9 (1.0–3.3)**	1.4 (0.7–2.5)
waist			1.0 (0.9–1.0)			**1.0 (1.0–1.0)**
education			0.9 (0.6–1.3)			0.6 (0.3–1.1)
smoking			0.8 (0.5–1.2)			1.4 (0.5–3.2)
History of HTN			**1.4 (1.0–2.1)**			1.6 (0.9–2.8)
DM			**2.0 (1.3–3.1)**			**2.1 (1.1–3.7)**
Family history of CV events			1.3 (0.8–2.1)			1.2 (0.6–2.2)

Model 1: unadjusted

Model 2: adjusted for age

Model 3: adjusted for age, WC, smoking, education, history of HTN, DM, and family history of CV events.

CKD chronic kidney disease, PRL prolactin, HTN hypertension, DM diabetes mellitus, CV cardiovascular

Bold values indicate statistical significance.

Interaction analyses revealed no statistically significant interaction between CKD and PRL on the odds of CV events outcome across all three models. This consistent pattern was observed regardless of gender. Model 3, the fully adjusted model, demonstrated that each one-unit increase in PRL did not significantly alter the odds of CV events in men with a history of CKD compared to those without (OR: 0.9; 95% CI: 0.9–1.0). Similarly, women with a history of CKD showed no association between each one-unit increase in PRL and lower odds of CV events compared to women without a history of CKD (OR: 0.9; 95% CI: 0.9–1.0). We re-examined the association by categorizing PRL and performing a sensitivity analysis that included individuals with a history of CVD. The results confirmed these findings, with detailed information provided in Supplementary Material.

## Discussion

The results of this population-based cohort study, with 20 years of follow-up, reveal that prolactin levels were not independently associated with CVD risk in either men or women across all models. CKD, however, was independently associated with an increased risk of CVD in both men and women, even after adjusting for potential confounding factors. Furthermore, no significant interaction was observed between prolactin levels and CKD in relation to CVD risk. These results suggest that prolactin levels may not substantially influence the association between CKD and CVD risk.

Prolactin is predominantly synthesized and secreted by lactotrophic cells in the anterior pituitary gland, although its expression is also observed in various tissues throughout the body. Its primary physiological function involves stimulating breast cell growth and differentiation, essential for lactation^4^. Beyond its role in lactation, prolactin plays a diverse role in several physiological processes, including insulin resistance, metabolic syndrome, inflammation, endothelial dysfunction, and atherosclerosis^4,18^.

Prolactin has both directly and indirectly affects the cardiovascular system. It regulates inflammation, promotes the development of vascular smooth muscle cell, facilitates the attachment of circulating mononuclear cells to the endothelium, which can lead to endothelial dysfunction^19^. Additionally, it impacts water and salt balance, potentially contributing to hypertension^20,21^. High levels of prolactin can also alter lipid profiles, promote weight gain and obesity^22,23^by stimulating hunger and reducing energy expenditure^24^. Furthermore, prolactin plays a role in insulin secretion, b-cell proliferation, and glucose metabolism^25–27^, although its effects in individuals with normal prolactin levels are still debated. Notably, prolactin’s association with peripartum cardiomyopathy (PPCM) is significant, as it may induce endothelial cell apoptosis, vasoconstriction, and impair cardiomyocyte function, potentially leading to PPCM^28^.

Chronic kidney Disease affects approximately 10% of the affects approximately 10% of the global population and is marked by an irreversible decline in renal function^29^. Despite its prevalence, managing CKD remains challenging, with CV events emerging as the leading cause of mortality across all stages^30,31^. The pathogenesis of CKD involves a complex interaction of factors, including volume overload, toxin retention, vascular calcification, and endothelial dysfunction, all contributing to accelerated atherosclerosis and left ventricular hypertrophy^1^. Additionally, CKD disrupts endocrine regulation, resulting to hormonal imbalances^32^, including elevated prolactin levels^33^. These elevations may arise from decreased kidney metabolism and increased secretion due to reduced dopamine levels in the uremic state^6,34^. While there is potential association between prolactin, CKD, and CV events, the precise mechanisms underlying these associations remain unknown. Despite some evidence suggesting that elevated prolactin levels may be associated with an increased risk of CV events, human studies in this field have yielded conflicting results. In a population-based study by Therkelsen et al. (2016)^35^which involved 3232 participants from the Framingham Heart Study, no significant association was observed between prolactin levels and comprehensive panel of incident cardiovascular disease risk factors. Consequently, they concluded that measuring prolactin in the community is unlikely to offer substantial insight into cardiometabolic risk. In another study involving 414 postmenopausal women, Amirzadegan et al. (2019)^36^reported no statistically significant correlation between the serum prolactin levels and the extent of coronary artery atherosclerosis, as measured by the Gensini score. Consistent with our study, Reuwer et al. (2009)^12^in a population-based study found that high levels of prolactin in the bloodstream did not predict future CV events among middle-aged individuals. Conversely, Haring et al. (2014)^11^in another population-based study observed a positive association between serum prolactin and cardiovascular mortality in both men and women. This contradictory finding may be partly attributed to differences in study design, duration of observation, definition of CV events, as well as the gender and age distribution of the study participants. To our knowledge, only been one study has investigated the association between prolactin and CKD regarding CV events risk^13^. This study followed 457 non-dialyzed CKD patients, including 229 males and 228 females, over an average of 38 months to monitor CV events. CV events was defined as a medical history of heart-related issues, symptoms of intermittent claudication and transient ischemic attack, and artery stenosis above 60% in imaging, as well as revascularization and amputation. The median age of patients for both genders was 52 years old. During the study period, there were 40 cardiovascular-related deaths and an additional 106 non-fatal CV events. Results showed that elevated prolactin levels in both male and female CKD patients were associated with an increased risk of cardiovascular events. Furthermore, for each 10 ng/mL increase in serum prolactin concentration, there was a 27% higher risk of experiencing a cardiovascular event.

The discrepancy in findings outcomes between our study and Carerro’s study, may potentially related to the variations in the age distribution of participants in the two studies. In our study, the subjects were relatively younger, which might have resulted in a lower incidence of CV events at earlier ages. This reduced likelihood of CV events occurring at younger ages could have masked the potential effect of prolactin in increasing the risk of CV events, particularly among individuals with CKD^37^. Additionally, our study focused on individuals with milder stages of CKD, which could influence the interaction between prolactin and CKD concerning future CV events risk. Moreover, differences in research objectives and study design may contribute to the contrasting findings. For example, Haring’s study^11^, aimed to establish a relationship between serum prolactin levels and cardiovascular mortality risk, whereas our study excluded participants with pre-existing CV events and focused on assessing the incidence of future CV events risk as the primary outcome. Furthermore, variations in the duration of follow-up may also play a role in the observed differences.

It should be noted that in this study, we excluded participants with hyperprolactinemia (PRL levels ≥ 100 ng/mL) to minimize the potential confounding effects of extreme prolactin elevations. However, a significant subset of participants (*n* = 202) exhibited prolactin levels between 30 and 100 ng/mL. Since prolactin levels above 30 ng/mL may still be clinically relevant, with potential implications for cardiovascular risk, excluding this group would have further reduced the statistical power of our analyses. Future research should explore the impact of different prolactin thresholds to more precisely define the role of moderate elevations in cardiovascular risk.

Our study has numerous strengths. The population-based nature enhances the generalizability of the findings to other population. Additionally, the long-term follow-up period and actively assessment of outcomes provided a more complete picture of the phenomenon under investigation. Furthermore, the study incorporates a thorough evaluation of potential confounding variables. As well, the minimal intra-assay variability in our data is likely due to all laboratory measurements being conducted at the same facility using a consistent assay method. However, our research also has limitations that warrant acknowledgment. Firstly, our participants are relatively young, which might have contributed to a lower incidence of CV events cases compared to an older population, potentially limiting the generalizability of our findings to elderly individuals. Further, the sample size of this study was small, particularly for detecting interactions, which may require larger cohorts to achieve sufficient statistical power. Although the interaction between CKD and prolactin was not statistically significant, but this study offers valuable preliminary insights into their relationship, which could inform future research. Additionally, measuring prolactin levels only at the study’s outset without considering variations over time might not fully representatively changes in prolactin levels throughout the study duration. While a single measurement is acceptable under certain conditions according to clinical practice guidelines^38^, the lack of serial measurements could limit the accuracy of our findings. In addition, the insufficient number of CKD occurrences did not let us to perform subgroup analysis based on the different CKD stages, which could have provided further insights. Further, although the analysis adjusted for multiple covariates, including age, WC, smoking status, education level, history of HTN, CVD, and DM, and family history of CV events, the lack of lipid levels as covariates remains a potential limitation. Finally, we excluded participants with prolactin levels ≥ 100 ng/mL, as such elevated levels may indicate pathological conditions that require further clinical and subclinical evaluation. As a population-based study, we were unable to conduct these assessments, which may have limited our ability to fully evaluate the association between prolactin levels and CVD events, particularly at the upper end of the prolactin range.

In conclusion, based on population-based data collected over a median follow-up period of 20 years, our study showed that prolactin levels were not independently associated with CVD risk in either men or women. CKD, however, was independently associated with an increased risk of CV events in both men and women. Notably, this elevated risk may not be substantially influenced by prolactin levels. Further investigation is warranted to confirm these findings.

## Statement of ethics

The ethics statement was issued by Shahid Beheshti University of Medical Sciences with approval number: IR.SBMU.ENDOCRINE.REC.1402.027. The consent was obtained both written and oral from each participant. In addition, the present study was performed in accordance with the Declaration of Helsinki.

## Electronic supplementary material

Below is the link to the electronic supplementary material.

Supplementary Material 1

## Data Availability

The data can be made available on reasonable request to the corresponding author FRT via E-mail.
