# Supplementary material for: Prolactin levels and chronic kidney disease and the subsequent risk of cardiovascular events: A long term population based cohort study
Source: Sci Rep. 2025 Feb 28;15:7198. doi: 10.1038/s41598-025-87783-1 (PMC11871319; doi:10.1038/s41598-025-87783-1)
Supplement: Supplementary file 1 — Supplementary Material 1 [file 41598_2025_87783_MOESM1_ESM.docx]

Supplementary material

Supplementary 1. Relationship between PRL and CVD, incorporating the effect of CKD on this association in both women and men

| variables | Men | | | Women | | |
| --- | --- | --- | --- | --- | --- | --- |
|  | Model 1 | Model 2 | Model 3 | Model 1 | Model 2 | Model 3 |
|  | OR (95% CI) | OR (95% CI) | OR (95% CI) | OR (95% CI) | OR (95% CI) | OR (95% CI) |
| PRL | 1.0 (0.9-1.0) | 1.0 (0.9-1.0) | 1.0 (0.9-1.0) | 1.0 (0.9-1.0) | 1.0 (0.9-1.0) | 1.0 (0.9-1.0) |
| CKD |  | **3.5 (2.5-4.8)** | **2.5 (1.7-3.6)** |  | **3.6 (2.5-5.4)** | **2.2 (1.4-3.5)** |
| Age |  |  | 1.5 (0.9-2.6) |  |  | 1.4 (0.7–2.5) |
| waist |  |  | 1.0 (0.9-1.0) |  |  | **1.0 (1.0-1.0)** |
| education |  |  | 0.9 (0.6-1.3) |  |  | 0.6 (0.3-1.1) |
| smoking |  |  | 0.8 (0.5-1.2) |  |  | 1.4 (0.5-3.7) |
| History of HTN |  |  | 1.4 (0.9-2.1) |  |  | 1.6 (0.9-2.8) |
| DM |  |  | **2.0 (1.3-3.1)** |  |  | **2.1 (1.1-3.7)** |
| Family history of CV events |  |  | 1.3 (0.8-2.1) |  |  | 1.2 (0.6-2.2) |
| Model 1: unadjusted  Model 2: adjusted for CKD  Model 3: adjusted for CKD, age, WC, smoking, education, history of HTN, DM, and family history of CV events.  CKD chronic kidney disease, PRL prolactin, HTN hypertension, DM diabetes mellitus, CV cardiovascular  Bold values indicate statistical significance. | | | | | | |

Supplementary 2. The association by including prolactin (PRL) as a categorical variable

| variables | Women | | Men | |
| --- | --- | --- | --- | --- |
|  | unadjusted model | Multi adjusted model* | unadjusted model | Multi adjusted model* |
|  | OR (95% CI) | OR (95% CI) | OR (95% CI) | OR (95% CI) |
| CKD | **3.7 (1.2-11.7)** | 2.0 (0.5-7.3) | **3.8 (2.3-6.2)** | **2.7 (1.5-4.6)** |
| PRL (ng/mL) |  |  |  |  |
| Q1: 0.6 | ref | ref | ref | ref |
| Q2: 7.3 | 0.7 (0.3-2.0) | 0.6 (0.2-2.0) | 0.8 (0.5-1.3) | 0.6 (0.5-1.6) |
| Q3: 11.7 | 0.7 (0.2-1.8) | 0.7 (0.2-2.1) | 1.2 (0.7-2.0) | 1.6 (0.7-3.3) |
| Q4:18.6 | 0.9 (0.3-2.2) | 1.0 (0.3-3.0) | 1.3 (0.5-3.1) | 2.0 (0.7-6.0) |
| CKD*PRL |  |  |  |  |
| Q1: 0.6 | Ref | Ref | Ref | Ref |
| Q2: 7.3 | 1.3 (0.3-5.1) | 1.8 (0.4-8.0) | 1.4 (0.5-2.3) | 1.2 (0.5-2.6) |
| Q3: 11.7 | 1.0 (0.2-3.9) | 1.0 (0.2-4.9) | 0.5 (0.2-1.4) | 0.5 (0.2-1.6) |
| Q4:18.6 | 0.7 (0.2-2.9) | 0.9 (0.2-3.8) | 0.5 (0.1-1.7) | 0.3 (0.0-1.6) |
| Age |  | 1.4 (0.7-2.5) |  | 1.5 (0.9-2.6) |
| waist |  | **1.0 (1.0-1.0)** |  | 1.0 (0.9-1.0) |
| education |  | 0.6 (0.3-1.2) |  | 0.8 (0.5-1.3) |
| smoking |  | 1.4 (0.2-3.7) |  | 0.8 (0.5-1.2) |
| History of HTN |  | 1.6 (0.9-2.8) |  | **1.4 (1.0-2.1)** |
| DM |  | **2.1 (1.1-3.7)** |  | **2.0 (1.3-3.1)** |
| Family history of CV events |  | 1.2 (0.6-2.2) |  | **1.3 (0.0-0.0)** |
| Model 1: unadjusted  *Adjusted for age, WC, smoking, education, history of HTN, DM, and family history of CV events.  CKD chronic kidney disease, PRL prolactin, HTN hypertension, DM diabetes mellitus, CV cardiovascular  Bold values indicate statistical significance. | | | | |
